# Robot-assisted laparoscopic resection of giant left renal angiomyolipoma and multiple left retroperitoneal angiomyolipomas: a case report of lymphangioleiomyomatosis

**DOI:** 10.1186/s12894-025-01924-2

**Published:** 2025-11-07

**Authors:** Jianjia Huang, Junjie Bai, Jun Lin, Xiaoyan Li, Jianhui Chen

**Affiliations:** 1 Department of Urology, Fujian Medical University Union Hospital, Fuzhou, China; 2 The Graduate School of Fujian Medical University, Fuzhou, China; 3 Shengli Clinical Medical College of Fujian Medical University, Department of Urology, Fujian Provincial Hospital, Fuzhou University Affiliated Provincial Hospital, Fuzhou, China; 4 Department of Pathology, Fujian Medical University Union Hospital, Fuzhou, China

**Keywords:** Renal angiomyolipoma, Lymphangioleiomyomatosis, Chylothorax, Robotic-assisted, Laparoscopic

## Abstract

We report a young female patient with a giant left renal angiomyolipoma (AML) and multiple left retroperitoneal AML. After undergoing robotic-assisted laparoscopic surgery, the tumor was successfully removed cleanly while preserving the normal renal unit. On the fourth day after surgery, she developed respiratory symptoms, including chylothorax, and was diagnosed with sporadic lymphangioleiomyomatosis (LAM) combined with multiple AML. After receiving sirolimus and symptomatic treatment, the patient recovered and had no recurrence of the disease during the follow-up period. This case demonstrates the significant application of robot-assisted laparoscopic nephron-sparing surgery in the treatment of a giant renal angiomyolipoma (14.0 cm in diameter), highlighting its feasibility and effectiveness in managing complex multifocal disease while preserving renal function.

## Introduction

Lymphangioleiomyomatosis (LAM), also termed pulmonary lymphangioleiomyomatosis, is a rare multisystem neoplastic disorder of low-grade malignancy characterized by diffuse cystic degeneration in bilateral lungs. This disease predominantly affects women of reproductive age, with exceedingly rare occurrences in males and pediatric populations [1, 2]. The characteristic clinical manifestations of LAM typically include progressive dyspnea, recurrent pneumothorax, chylothorax, hemoptysis, and chest pain. According to guidelines published by the European Respiratory Society (ERS), LAM is classified into two subtypes: sporadic lymphangioleiomyomatosis (S-LAM) and tuberous sclerosis complex-associated LAM (TSC-LAM) [2]. The estimated incidence of S-LAM in adult females is approximately 1 in 400,000, while 30–40% of adult females with tuberous sclerosis complex (TSC) develop comorbid LAM.

Johnson, Franz et al., Urban et al., and Johnson [3–6]. Extrapulmonary manifestations of LAM include renal angiomyolipoma (AML; also termed vascular myolipoma) and retroperitoneal solid or cystic-solid lymphangioleiomyomas (synonymous with lymphatic leiomyomas). Studies have demonstrated that renal AML and LAM share histogenetic cellular components [7, 8]. In China, clinical case reports of LAM complicated by renal AML are exceedingly rare, resulting in insufficient awareness of this comorbidity, where underdiagnosis and misdiagnosis remain prevalent. Furthermore, limited therapeutic options are currently available. We present a case of a young female with giant renal AML who developed chylothorax following surgical intervention, ultimately diagnosed with S-LAM concomitant with renal AML and managed with pharmacotherapy. This report is supplemented by a comprehensive literature review addressing the pathophysiological and clinical associations between these two disease entities.

## Case presentation

**Fig. 1:**
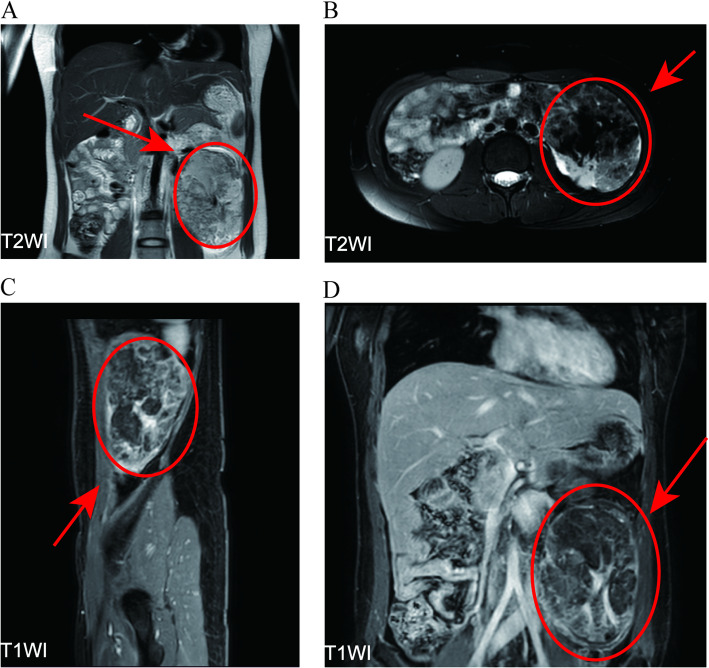
Pre-surgical MRI images. (**A**: T2WI, Frontal plane. **B**: T2WI, Axial section. **C**: T1WI, Sagittal plane. **D**: T1WI, Frontal plane)

**Fig. 2:**
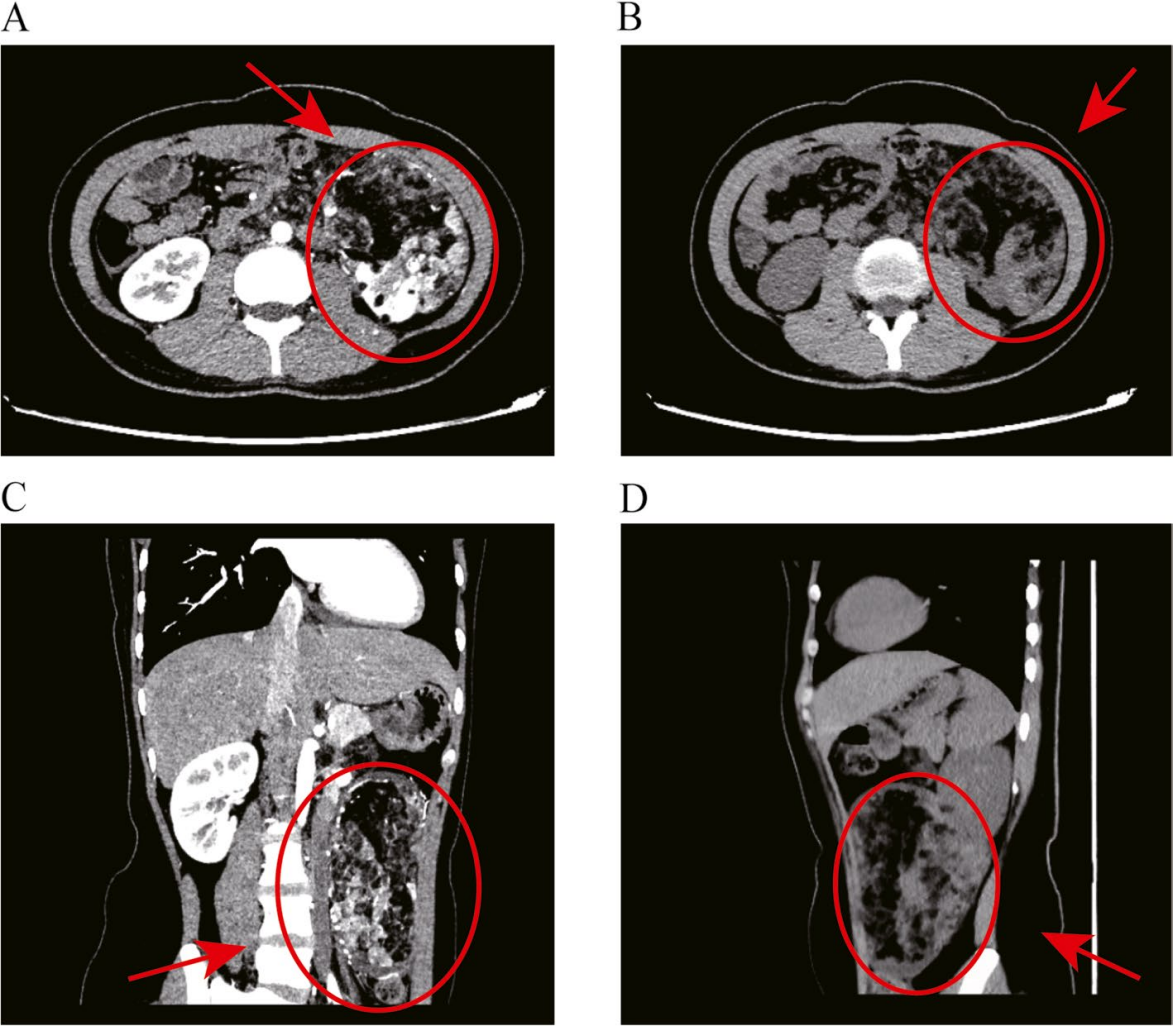
Pre-surgical CT scan. (**A**: CT Enhancement Scan, Axial section. **B**: CT Scan, Axial section. **C**: CT Enhancement Scan, Frontal plane. **D**: CT Scan, Sagittal plane)

**Fig. 3:**
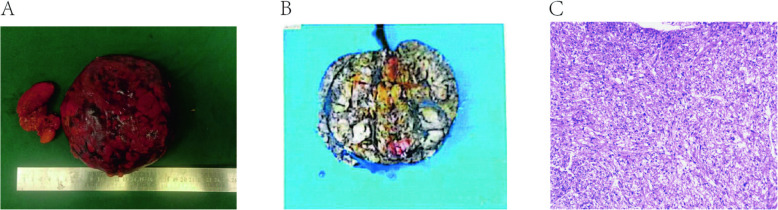
Tumor histopathology images. (**A**-**B**: Macroscopic of tumor. **C**: Microscopic of tumor)

A 25-year-old female presented to our outpatient clinic with a left renal mass incidentally detected during routine physical examination. MRI revealed multiple hamartomatous lesions in the left renal lower pole, retroperitoneal left renal hilum, perirenal fascia, left retrocrural space, and para-aortic region, along with multiple left renal hamartomas (Fig. 1). Subsequent CT renal arteriography during urology admission demonstrated: 1) a left renal lower pole angiomyolipoma (AML) supplied by the left anteroinferior renal artery and aortic branches; 2) multiple retroperitoneal AMLs near the left renal hilum and retrocrural space; 3) additional small left renal AMLs; and 4) hepatic fatty infiltration, with normal right renal findings (Fig. 2). Physical examination revealed a large firm abdominal mass confined to the left hemiabdomen. Preoperative laboratory parameters (hematologic, hepatic/renal, cardiac enzymes) were unremarkable. Chest radiographs showed no abnormalities, and the patient denied any respiratory symptoms. The patient underwent successful robot-assisted laparoscopic left partial nephrectomy with retroperitoneal tumor resection via transperitoneal approach, achieving complete excision with 20-min renal artery warm ischemia and < 100 mL blood loss. Postoperative recovery was notable for early ambulation (day 1), minimal drainage (100 mL/day), and drain removal by day 3, with preserved renal function. Histopathology confirmed AML (14.0 × 12.5 × 8.5 cm) showing characteristic immunohistochemical profile: SMA + smooth muscle components, focal MelanA +, scant HMB45 +, S100 + adipocytes, CD34 + vasculature, TFE3-, and Ki67 ~ 3% proliferation index (Fig. 3).

**Fig. 4:**
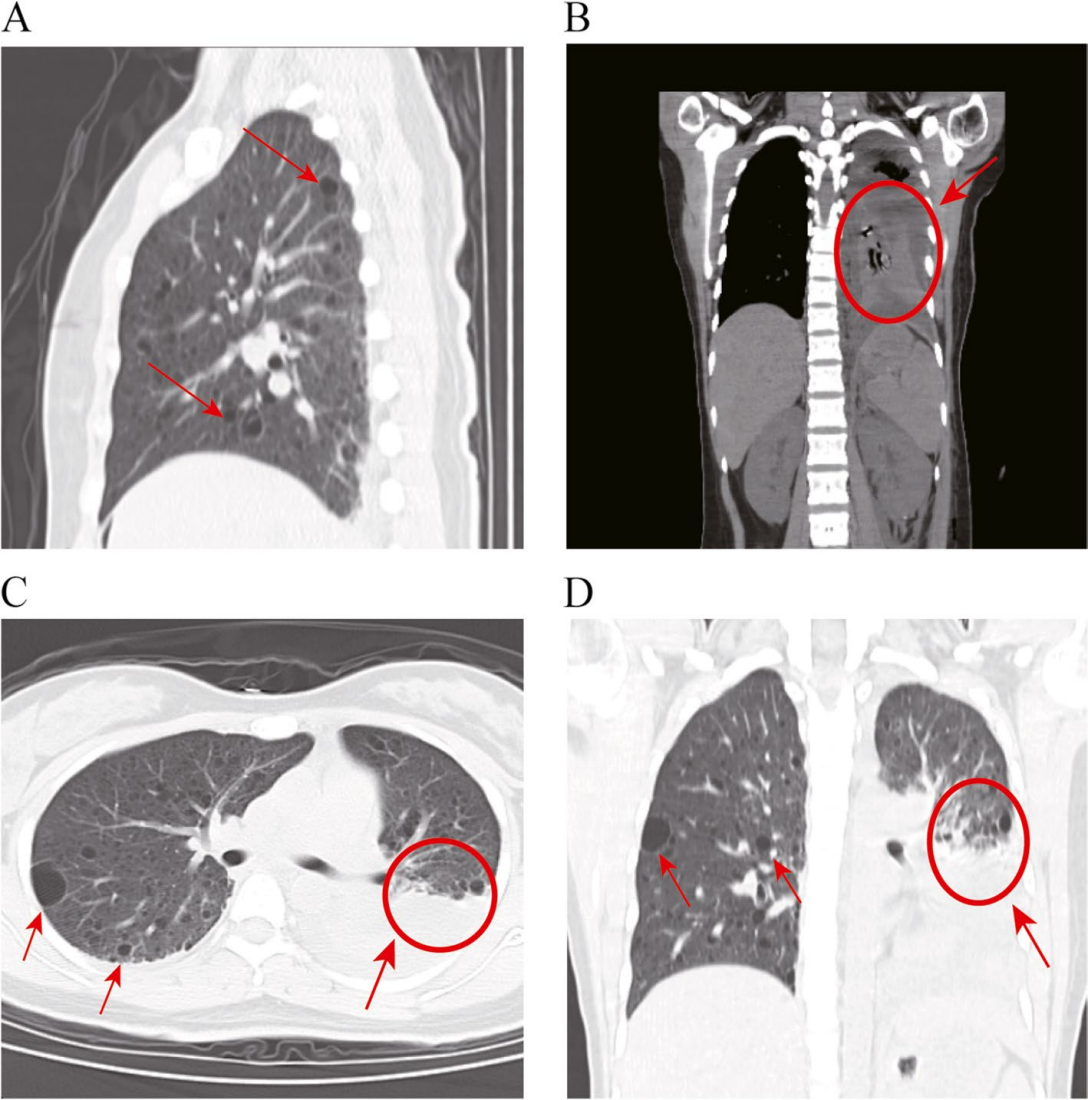
Pulmonary CT images

On postoperative day 4, the patient developed chest tightness and dyspnea. CT imaging revealed bilateral pleural effusions (left predominant), bilateral lower lobe pneumonitis with left lower lobe consolidation, and diffuse pulmonary cystic lesions suggestive of pulmonary lymphangioleiomyomatosis (LAM). Ultrasound-guided left thoracentesis drained 200 mL/day of chylous effusion (chylomicron +, Rivalta test 3 +, RBC 3 +) (Fig. 4). Comprehensive diagnostic evaluation – including fundoscopy, brain MRI, echocardiography, and TSC1/2 genetic testing (negative for mutations) – confirmed sporadic lymphangioleiomyomatosis (LAM) with giant renal angiomyolipomas (AMLs). The patient was managed with sirolimus (mTOR inhibitor), low-fat diet, and thoracic drainage, achieving clinical resolution by day 10. Twelve-month follow-up demonstrated sustained remission without recurrence or complications.

## Discussion

Current evidence indicates that lymphangioleiomyomatosis (LAM) arises from TSC1/2 gene mutations driving constitutive activation of the mTOR signaling pathway, which promotes dysregulated proliferation of neoplastic LAM cells. This pathognomonic mechanism underlies its classification as a systemic neoplastic disorder, characterized by progressive structural destruction and functional impairment in both pulmonary and extrapulmonary organ systems [1, 9]. Both lymphangioleiomyomatosis (LAM) and angiomyolipoma (AML) belong to the neoplastic family of perivascular epithelioid cell tumors (PEComas). Renal AMLs are present in nearly 100% of TSC-LAM patients (tuberous sclerosis complex-associated LAM) and approximately 50% of S-LAM cases (sporadic LAM), reflecting their shared pathogenic origin within this tumor spectrum [10]. According to the 2017 American Thoracic Society/Japanese Respiratory Society (ATS/JRS) diagnostic criteria for lymphangioleiomyomatosis (LAM), our patient's diagnosis was confirmed through histopathological verification of renal and retroperitoneal angiomyolipomas (AMLs), postoperative development of chylothorax, thoracic CT evidence of bilateral diffuse thin-walled cystic lesions, and exclusion of tuberous sclerosis complex (TSC) via negative TSC1/2 genetic testing coupled with absence of extracutaneous TSC manifestations. This comprehensive evaluation established the definitive diagnosis of pulmonary LAM with concomitant renal and retroperitoneal AMLs, consistent with sporadic LAM (S-LAM) under the ATS/JRS classification system [11]. Case reports of LAM combined with multiple extrapulmonary AML in the kidney, liver, retroperitoneum, etc., are rare [12], patient showed this on imaging as well. Renal AML is usually benign, although rare cases with metastasis have been reported. In the present case, the resected specimen demonstrated typical benign histology without evidence of malignant transformation or metastasis to distal [13]. LAM also has metastatic potential, and LAM cells can be found in enlarged lymph nodes, blood, and other body fluids [14–16], thus, distant metastasis seems to be the most likely explanation for this case, but whether the primary focus is renal AML or pulmonary LAM needs to be further explored.

Most renal AMLs are incidentally detected in asymptomatic patients. Current guidelines recommend conservative management (e.g., observation) for lesions < 4 cm in diameter. Therapeutic interventions (embolization, partial nephrectomy) are typically advised for tumors exceeding 4 cm, particularly when accompanied by clinical symptoms (flank pain, hematuria) or high-risk features. Notably, renal AMLs > 4 cm containing intratumoral aneurysms ≥ 5 mm carry a significantly elevated risk of spontaneous rupture with hemorrhage (estimated annual risk 10–15%), necessitating proactive intervention to prevent life-threatening complications [17]. Given the benign nature of renal angiomyolipomas (AMLs), nephron-sparing interventions such as selective arterial embolization or partial nephrectomy are prioritized to preserve renal function. Although no universal threshold exists, AMLs exceeding 10 cm in maximal diameter are conventionally defined as ‘giant’ [18]. Robot-assisted laparoscopic nephrectomy has been proven in previous studies to be a safe and effective method for treating renal AMLs smaller than 10 cm [19–21]. As for exceptionally giant AMLs with extensive renal parenchymal involvement, where complete resection poses significant technical challenges, traditional radical nephrectomy was historically employed. Modern advances in surgical techniques (e.g., robotic-assisted approaches, super-selective embolization) and perioperative management now allow nephron preservation even in complex cases, reserving nephrectomy for irreparable vascular damage or life-threatening hemorrhage [22, 23]. While nephrectomy significantly impacts renal functional reserve, nephron-sparing tumor resection remains an alternative after comprehensive evaluation. Treatment for giant renal AML larger than 10 cm is usually reported as a case report, and open surgery or laparoscopic surgery is used [23, 24]. This case represents the documented application of robot-assisted laparoscopic nephron-sparing surgery for a giant renal AML (14.0 cm), exceeding the maximum tumor diameter (11.0 cm) reported in previous robotic series. The transperitoneal approach provided enhanced maneuverability for complete tumor resection while achieving optimal renal preservation (renal artery warm ischemia time: 20 min; blood loss < 100 mL). Notably, this technique enabled simultaneous radical excision of both the renal AML and multiple retroperitoneal lesions, demonstrating the robotic platform's efficacy in managing complex multifocal AML.

The incidence of chylothorax varies among different types of surgeries, but it is more common in surgeries involving lymph node dissection or extensive tissue removal, as these procedures may damage the thoracic duct, leading to chylothorax [25, 26]. Reports of pleural effusion and chylothorax after the surgical removal of a giant renal angiomyolipoma are extremely rare. This may be associated with lymphangioleiomyomatosis (LAM), especially in young female patients, as LAM can cause the dilation and dysfunction of lymphatic vessels, leading to the accumulation of lymphatic fluid and the formation of chylothorax. In some LAM patients, pleural effusion occurs due to lymphatic lesions on the diaphragm, resulting in communication between the thoracic and abdominal cavities [27]. Additionally, the possibility of surgery-related factors cannot be ruled out. During the procedure, damage to the pleura and lymphatic vessels may occur, leading to the development of post-operative pleural effusion. Moreover, post-operative fluid balance and lymphatic drainage may be affected, thereby increasing the risk of pleural effusion [28]. The occurrence of chylothorax not only affects the post-operative recovery of patients but may also lead to serious respiratory, immune, and metabolic complications. Therefore, early identification and effective management of chylothorax are crucial for improving patient prognosis. Sirolimus, an mTOR (mammalian target of rapamycin) inhibitor, is the first FDA-approved drug for treating LAM (lymphangioleiomyomatosis). It has been shown to improve lung function, blood oxygen levels, exercise capacity, and quality of life in LAM patients [29].Currently, Sirolimus has not been approved in China, so reports on its use for treating LAM (Lymphangioleiomyomatosis) and AML (Angiomyolipoma) cases in the country are still limited. While Sirolimus has shown positive effects in treating lung function and AML in LAM patients, most patients exhibit a rebound in kidney AML and lung function, approaching baseline levels, within a year after discontinuing Sirolimus treatment. This indicates that Sirolimus does not provide a curative treatment. However, in cases where patients diagnosed with LAM received Sirolimus treatment and were followed up for one year, no tumor recurrence or symptoms of LAM were observed.

Retrospectively, a more comprehensive preoperative evaluation—including chest CT and serum VEGF-D—could have identified early-stage LAM and potentially allowed mTOR-inhibitor therapy to reduce AML volume, possibly obviating the need for immediate surgery [30, 31]. However, the absence of pulmonary symptoms and the routine chest X-ray being read as normal led to this opportunity being missed. To avoid misdiagnosis of LAM, clinicians should remain alert to its extrapulmonary manifestations—especially renal AML and retroperitoneal lymphangioleiomyomas. A complete work-up (contrast-enhanced CT of chest–abdomen–pelvis, brain MRI for TSC stigmata, and TSC1/2 gene testing) is warranted in any reproductive-age woman presenting with AML ≥ 4 cm. Postoperative surveillance must include prompt evaluation of new respiratory symptoms to detect complications such as chylothorax at an early stage.

## Conclusion

The use of Da Vinci robotic-assisted laparoscopic surgery in the treatment of renal angiomyolipomas (AML) and retroperitoneal multiple lesions has shown excellent results, particularly in younger patients with complex lesions. Minimally invasive surgery helps reduce trauma and shorten recovery time. In the case of this patient, LAM was detected early post-surgery and treated promptly, with Sirolimus further controlling the disease progression. In future clinical management, a more comprehensive systemic evaluation of patients with renal angiomyolipomas should be emphasized, especially early screening for LAM, to improve long-term survival rates and quality of life for patients.

## Data Availability

The original contributions presented in the study are included in the article/Supplementary material.
